# Synergistic attenuation of complete freund’s adjuvant-induced inflammation in mice using shinbaro-pelubiprofen: a novel therapeutic complex

**DOI:** 10.1186/s10020-025-01083-y

**Published:** 2025-01-21

**Authors:** Hyunseong Kim, Jin Young Hong, Wan-Jin Jeon, Hyun Kim, Changhwan Yeo, Junseon Lee, Yoon Jae Lee, In-Hyuk Ha

**Affiliations:** Jaseng Spine and Joint Research Institute, Jaseng Medical Foundation, Gangnamdae-ro 540, Seoul, 135-896 Republic of Korea

**Keywords:** Complete freund’s adjuvant, Inflammation, Macrophages, Pain, Pelubiprofen, Shinbaro

## Abstract

**Background:**

Inflammation is a critical protective response in the body, essential for combating infections and healing injuries. However, chronic inflammation can be harmful and significantly contribute to the development and progression of chronic diseases, with macrophage-mediated responses being central to these processes. This study presents “SBR-Pel,” a new therapeutic blend of Shinbaro tab (SBR), a traditional herbal formula, and pelubiprofen (Pel), a non-steroidal anti-inflammatory drug, and investigated their combined anti-inflammatory effects to create a treatment that both improves efficacy and reduces side effects.

**Methods:**

To this end, we performed both in vitro and in vivo analyses, utilizing a mouse model of inflammation. Viability and cytotoxicity assays, immunohistochemistry, enzyme-linked immunosorbent assays, real-time polymerase chain reaction assays, nociception assays, writhing tests, and blood biochemical analyses were performed.

**Results:**

In vitro, SBR-Pel synergistically reduced the production of nitric oxide and reactive oxygen species and the expression of pro-inflammatory cytokines. SBR-Pel also significantly attenuated paw edema in vivo in a Complete Freund’s adjuvant-induced inflammation model in adult mice. Furthermore, immunohistochemical analyses showed that treatment with SBR-Pel reduced both the infiltration of CD68^+^ macrophages and the expression of pro-inflammatory cytokines in inflamed tissues. Additionally, compared with individual treatment alone, SBR-Pel enhanced the expression of anti-inflammatory cytokines *CD206*, *TGF-β*, and *IL-10*, indicating a synergistic effect. Our research demonstrates that SBR-Pel effectively diminishes inflammatory pain by reducing macrophage infiltration and pro-inflammatory cytokine secretion. Additionally, while 1.5 mg/kg of Pel alone increases levels of liver and kidney toxicity markers, such as aspartate aminotransferase, alanine aminotransferase, and creatinine, combining it with SBR at a reduced dosage of 0.5 mg/kg maintains these markers at normal levels.

**Conclusions:**

This combined effect highlights SBR-Pel’s potential as an effective treatment for inflammatory diseases driven by heightened macrophage activity, while also minimizing side effects by reducing the Pel dosage.

**Supplementary Information:**

The online version contains supplementary material available at 10.1186/s10020-025-01083-y.

## Background

Inflammation is an essential physiological response that serves as the cornerstone of the defense mechanisms in the body. It plays a crucial role in combating infections and aiding the healing process following injury. Despite its protective functions, prolonged or chronic inflammation can lead to deleterious effects that significantly contribute to the initiation and progression of various chronic diseases (Chen et al. 2018; Michels da Silva et al. 2019). Central to this adverse process are macrophage-mediated responses, which are pivotal for regulating the inflammatory milieu (Austermann et al. 2022). The extended or excessive activity of M1 macrophages can generate reactive oxygen species (ROS) and nitric oxide (NO), contributing to chronic inflammation and tissue damage, such as atherosclerosis, rheumatoid arthritis, and systemic lupus erythematosus (Park 2021; Yang et al. 2023). Therefore, the balance between M1 and M2 macrophages is vital for health, allowing the body to mount an effective defense against pathogens and injury, ensuring the resolution of inflammation, and promoting tissue repair (Yang et al. 2023; Viola et al. 2019; Xia et al. 2023).

The complex interplay between the various cellular and molecular components of the inflammatory response underscores the need for therapeutic interventions that can effectively modulate these processes (Yoshinaga and Takeuchi 2024). Combining natural products and pharmaceuticals can offer a multifaceted treatment approach, which is often effective in treating complex diseases. This strategy encourages innovation, addresses the pressing issue of drug resistance, enhances drug efficacy while potentially reducing side effects, and promotes a holistic and sustainable healthcare approach (Bulaj et al. 2016; Cheon and Ko 2022; Yang and Wang 2021). Shinbaro tab (SBR), a complex herbal composition extracted from Achyranthes japonica (Gucheok), Saposhnikovia divaricata (Bangpung), Eucommia ulmoides (Useul), Acanthopanax sessiliflorus (Ohgapi), Taxillus chinensis (Duchung), and Glycine max (Heukdu), has been shown in previous studies to have anti-inflammatory and pain-relieving effects. It works by reducing the activity of inflammation-causing molecules, such as inducible nitric oxide synthase (iNOS), cyclooxygenase-2 (COX-2), and tumor necrosis factor (TNF)-α, slowing down cartilage damage, and inhibiting matrix metalloproteinase (MMP)-2 and MMP-9 in rats with arthritis. SBR is reportedly as effective as celecoxib, a drug that selectively inhibits COX-2, but with a better safety profile in clinical trials (Lee et al. 2011). Additionally, Pelubiprofen (Pel) is a nonsteroidal anti-inflammatory drug (NSAID) and a COX-2 inhibitor used in the treatment of conditions such as osteoarthritis, rheumatoid arthritis, and lower back pain. Studies have shown that Pel effectively reduces inflammation in rat paws (measured by volume, width, and thickness) and alleviates pain by blocking the production of prostaglandin E2 (Shin et al. 2011; Son et al. 2023; Yoon et al. 2023).

In this context, our study investigated “SBR-Pel,” a novel therapeutic formulation that combines SBR, a traditional herbal mixture used as an analgesic and anti-inflammatory drug for osteoarthritis, and Pel, a non-steroidal anti-inflammatory drug (NSAID). Specifically, we investigated the mechanism of the synergistic anti-inflammatory effect of SBR-Pel, with a particular focus on its effect on macrophage-mediated inflammatory responses in RAW 264.7 macrophages and in mice with Complete Freund’s adjuvant (CFA)-induced inflammation.

## Methods

### Preparation of shinbaro and pelubiprofen tablets

Shinbaro tablets (300 mg/tablet) were purchased from GC Biopharma Corp. (Yongin, Korea), and pelubiprofen tablets (30 mg/tablet) were obtained from Daewon Pharmaceuticals Co., Ltd. (Seoul, Korea) for use in in vitro and in vivo experiments.

### RAW264.7 cell culture and treatment

RAW264.7 macrophages were seeded at the appropriate densities on various cell culture plates designated for the different assays and cultured in Dulbecco’s Modified Eagle Medium enriched with 10% heat-inactivated fetal bovine serum, 100 µg/mL streptomycin, and 100 U/mL penicillin (Kim et al. 2023). The cells were incubated at 37 °C under a humidified atmosphere consisting of 5% CO_2_ and 95% air. The day following seeding, the cells were treated with 200 µg/mL SBR and either 10 or 50 µM Pel. At 30 min after administering these drugs, the cells were exposed to 1 µg/mL of lipopolysaccharide (LPS) and incubated for 24 h. Then, both the supernatant and cells were collected for subsequent analysis. The details of our experimental timeline are presented in Scheme 1.

Scheme 1. Schematic timeline of RAW264.7 macrophage culture and drug treatment for in vitro experimental analysis.

### In vitro cell viability assays

Cell viability was determined using the Cell Counting Kit (CCK)-8 assay (Dojindo, Kumamoto, Japan). First, to find the optimal concentrations that reduce NO without causing cytotoxicity, we measured cell viability by treating cells with various concentrations of SBR (0–800 µg/mL) and Pel (0-200 µM) without LPS stimulation using the CCK assay. Next, to evaluate the effects of SBR and Pel in the presence of LPS, the cells were classified into several groups: the blank (no-treatment) group; 1 µg/mL LPS-treated group (LPS group); 200 µg/mL SBR pretreated + LPS group (SBR group); 10 or 50 µM Pel pretreated + LPS groups (Pel groups); and the 200 µg/mL SBR and 10 µM Pel pretreated + LPS group (SBR + Pel group). To perform the analysis, 2 × 10^4^ RAW264.7 macrophages were seeded in each well of a 96-well plate and cultured for 24 h after treating with the drugs under two conditions: with or without LPS. CCK-8 solution was added to the culture medium at a ratio of 10% v/v. At 4 h post-treatment, the absorbance of each well was recorded at 450 nm using a BioTek Epoch microplate reader (BioTek, Winooski, VT, USA). Cell viability was determined by dividing the absorbance of the treated cells by that of the control cells and multiplying the quotient by 100 to yield a percentage.

### In vitro cytotoxicity assays

NO and ROS assays were performed to determine the cytotoxic effects of SBR-Pel. For the NO production assay, we seeded 2 × 10^**4**^ RAW264.7 macrophages in each well of 24-well plates and treated them with various drug combinations and 1 µg/mL of LPS for 24 h. After incubation, 50 µL of the culture supernatant was transferred to a new 24-well plate. Equal volumes of Griess reagents A and B were added to the culture supernatant. The absorbance was measured at 540 nm using a microplate reader, with fresh culture medium serving as a control in all experiments. The concentration of nitrite, which is indicative of NO production, was quantified by comparison with a standard curve of sodium nitrite. We also analyzed the cell lysates to assess ROS generation using flow cytometry. RAW 264.7 cells were seeded at 2 × 10^4^ cells/well in six-well plates and cultured at 37 °C for 24 h. After incubation, the cells were treated with 200 µg/mL SBR and 10 or 50 µM Pel. After 24 h of treatment, the cells were incubated with 10 µM of the cell-permeable fluorogenic probe, 2′,7′-dichlorodihydrofluorescein diacetate (DCFDA; Sigma-Aldrich, St. Louis, MO, USA), for 30 min at 37 °C to stain the ROS. DCFDA fluorescence was measured using a spectrofluorometer (BD Biosciences, Franklin Lakes, NJ, USA) with excitation and emission wavelengths set at 484 and 530 nm, respectively.

### Immunocytochemistry

RAW264.7 macrophages were seeded at a density of 1 × 10^4^ cells per well in 24-well plates and subjected to drug and LPS treatments following the experimental timeline specified in Scheme 1. Subsequently, the cells were subjected to fixation with 4% paraformaldehyde for 30 min, followed by three 5-min washes with phosphate-buffered saline (PBS; Gibco BRL, Grand Island, NY, USA). The cells were permeabilized in PBS containing 0.2% Triton X-100 for 5 min, followed by two PBS washes for 5 min each, and blocking with 2% normal goat serum (NGS) prepared in PBS for 1 h. Primary antibodies against iNOS (1:100, Abcam, Cambridge, UK) and CD68 (1:500; Millipore, Burlington, MA, USA) were applied, diluted in 2% NGS, and left to incubate overnight at 4°C. After three 5-min washes with PBS, fluorescent secondary antibodies (FITC-conjugated goat anti-mouse, rabbit immunoglobulin G (IgG), or rhodamine-conjugated goat anti-mouse, rabbit IgG, all from Jackson ImmunoResearch Labs) were applied at a 1:300 dilution in 2% NGS for 2 h at room temperature. Following three 5-min washes with PBS, staining with 4’,6-diamidino-2-phenylindole (DAPI, 1:1000, TCI, Tokyo, Japan) was performed. Finally, the samples were mounted using fluorescence mounting medium (DAKO, Carpinteria, CA, USA) and confocal microscopy (Eclipse C2 Plus, Nikon, Tokyo, Japan) was used to acquire 10 representative images at 200× magnification, utilizing the same acquisition settings. The average fluorescence intensity was quantitatively analyzed using ImageJ software (National Institutes of Health, Bethesda, MD, USA).

### Enzyme-linked immunosorbent assay (ELISA)

Expression levels of the pro-inflammatory markers interleukin (IL)-6 and TNF-α in culture supernatants from drug-treated RAW264.7 macrophages were evaluated using ELISA kits (BD Biosciences) following the manufacturer’s instructions.

### Animals

Adult C57BL/6 N male mice, aged 7 weeks and weighing 20–22 g, were obtained from Samtako (Osan, Korea) and housed in a pathogen-free facility. The facility maintained a temperature of 25 °C, relative humidity of 50%, and a constant 12-h light-dark cycle. All animal experimental procedures were approved by the Jaseng Animal Care and Use Committee (Approval Number: JSR-2024-01-003-A). To ensure objectivity, all experiments were conducted by investigators who were blinded to the drug treatments during behavioral testing.

### CFA-induced mouse inflammation model and drug administration

Inflammation was triggered by administering 50 µL CFA (1 mg/mL; Sigma-Aldrich) via intraplantar injections into the right hind paws, with the procedure carried out under short-term anesthesia using 2% isofluorane (Forane; BK Pham, Goyang, Korea). The study involved six different experimental treatments: a Sham group receiving saline injections, a CFA group receiving CFA injections, an SBR group treated with 100 mg/kg SBR, Pel groups treated with either 0.5 or 1.5 mg/kg Pel, and a combined SBR + Pel group treated with 100 mg/kg SBR and 0.5 mg/kg Pel. Following the CFA injections, each treatment was provided orally in a single 100-µL bolus daily for 7 consecutive days. The experimental design and timeline of the procedure are presented in Scheme 2.

Scheme 2. Schematic timeline of CFA intraplantar injection and oral drug administration for in vivo experimental analysis.

### Measurement of paw thickness

Paw thickness was measured at different intervals (0, 1, 3, and 7 days) using an IP65 digital micrometer (Mitutoyo, Kawasaki, Japan). For each mouse, the thickness of each paw was measured thrice to determine the average thickness. Additionally, the final improvement rate for paw thickness was calculated using the average paw thickness measured on Day 7, applying the following formula, and presented in Table 1.

**Table 1:** Paw edema improvement after 7 days of oral drug administration of treatments in CFA-injected mice

	Improvement rate of paw edema (%)
SBR	9.61%
Pel_0.5	7.24%
Pel_1.5	18.38%
SBR-Pel	21.39%

CFA: Complete Freund’s Adjuvant; Pel: Pelubiprofen; SBR: Shinbaro

$$\eqalign{& {\rm{Improvement rate of paw thickness }}\left( {\rm{\% }} \right){\rm{ = }} \cr & \left( {{\matrix{{\rm{Average}}\>{\rm{paw}}\>{\rm{thickness}}\>{\rm{exp,}}\>{\rm{Day}}\>{\rm{7}} \hfill \cr {\rm{ - Average}}\>{\rm{paw}}\>{\rm{thickness}}\>{\rm{CFA,}}\>{\rm{Day}}\>{\rm{7}} \hfill \cr} \over {{\rm{Average}}\>{\rm{paw}}\>{\rm{thickness}}\>{\rm{CFA,}}\>{\rm{Day}}\>{\rm{7}}}}} \right)\; \times \;{\rm{100}} \cr} $$

Abbreviations: Average paw thickness exp, Day 7 represents the average paw thickness of the experimental group on Day 7, Average paw thickness CFA, Day 7 represents the average paw thickness of the CFA group on Day 7.

### Histology and immunohistochemistry

After perfusing the coronary artery with 0.9% saline for hematoxylin and eosin (H&E) and immunohistochemistry, the plantar skin from the right hindlimb, along with the liver, kidney, and lung, was carefully extracted and preserved in 4% paraformaldehyde at 4°C overnight. Subsequently, the specimens were dehydrated using a series of ethanol solutions, embedded in paraffin, and then sliced into 4-µm-thick sagittal sections. The sections were stained with H&E to assess dermal inflammation. Briefly, the sections were soaked in hematoxylin for 2 min and 30 s, washed under running tap water for 2 min, and stained with eosin for 50 s. After staining, the sections were rehydrated using an ethanol gradient (70–100%) and cleared in xylene. The stained samples were examined and photographed under a Nikon light microscope at 100× magnification using manual tile scanning to obtain the entire low magnification image of the paw. High magnification images of the footpads were captured at 40× magnification. The dermal area and thickness were quantified from the 100× scanned images using ImageJ software (National Institutes of Health). Additionally, the organ indices for the three tissues were calculated and compared using the formula: “Organ index = (organ weight ÷ mouse weight) × 100%.” The tissue injury scores for the liver, kidney, and lung were evaluated based on established references (Gori et al. 2019; Suzuki et al. 2011; Solez et al. 2008).

Immunohistochemistry was performed to investigate the inflammatory response using primary antibodies against CD68 (dilution 1:500; Millipore, Burlington, MA, USA). The tissue sections from each group were probed overnight with the primary antibody at 4°C. Then, the sections were washed three times with PBS before incubation with FITC-conjugated goat anti-mouse rabbit IgG secondary antibody. The stained sections were imaged at 100× magnification using the z-stack and tile scanning techniques with a confocal microscope (Eclipse C2 Plus, Nikon). Following the capture of images from seven animals in each group, the intensity of the CD68 signal was quantified using ImageJ (National Institutes of Health). Additionally, based on these values, the final improvement rate for CD68^+^ intensity was calculated using the following formula (Eq. 1), and the results are presented in Table 2.

**Table 2:** CD68+ intensity improvement rate after 7 days of oral drug administration in CFA-injected mice

	Improvement rate of CD68^+^ intensity (%)
SBR	17.94%
Pel_0.5	16.89%
Pel_1.5	27.63%
SBR-Pel	38.59%

CFA: Complete Freund’s Adjuvant; Pel: Pelubiprofen; SBR: Shinbaro

1 $$\eqalign{& {\rm{Improvement rate of CD6}}{{\rm{8}}^{\rm{ + }}}{\rm{intensity }}\left( {\rm{\% }} \right){\rm{ = }} \cr & \left( {{\matrix{{\rm{Average}}\>{\rm{CD68}}\>{\rm{intensity}}\>{\rm{exp,}}\>{\rm{Day}}\>{\rm{7 - }} \hfill \cr {\rm{Average}}\>{\rm{CD68}}\>{\rm{intensity}}\>{\rm{CFA,}}\>{\rm{Day}}\>{\rm{7}} \hfill \cr} \over {{\rm{Average}}\>{\rm{CD68}}\>{\rm{intensity}}\>{\rm{CFA,}}\>{\rm{Day}}\>{\rm{7}}}}} \right)\; \times \;{\rm{100}} \cr} $$

Abbreviations: Average CD68 intensity exp, Day 7 represents the average CD68 intensity of the experimental group on Day 7, Average CD68 intensity CFA, Day 7 represents the average CD68 intensity of the CFA group on Day 7.

### Nociception assay

Sensory evaluation was conducted during drug administration for 7 days. The hotplate test is a widely used method for assessing pain sensitivity to thermal exposure. This test was conducted with reference to previous research studies. In this study, the assessment was conducted four times, on Days 0, 1, 3, and 7, during the week following the initiation of drug therapy and administration of CFA, as well as prior to CFA injection. Briefly, the animals were positioned on a surface that had been heated to a steady temperature of 55 °C, and the duration for which the animals displayed a pain response (paw licking or jumping) was recorded. Each participant underwent this test four times, and the average of these trials was presented as the final result. Additionally, the final improvement rate for thermal pain on Day 7 was calculated using the following formula (Eq. 2). The results are presented in Table 3.

**Table 3:** Thermal pain improvement rate after 7 days of oral drug administration in CFA-injected mice

	Improvement rate of thermal pain (%)
SBR	20.16%
Pel_0.5	13.65%
Pel_1.5	51.25%
SBR-Pel	49.53%

CFA: Complete Freund’s Adjuvant; Pel: Pelubiprofen; SBR: Shinbaro

2 $$\eqalign{& {\rm{Improvement rate of thermal pain }}\left( {\rm{\% }} \right){\rm{ = }} \cr & \left( {{\matrix{{\rm{Average}}\>{\rm{latency}}\>{\rm{exp,}}\>{\rm{Day}}\>{\rm{7 - }} \hfill \cr {\rm{Average}}\>{\rm{latency}}\>{\rm{CFA,}}\>{\rm{Day}}\> \hfill \cr} \over {{\rm{Average}}\>{\rm{latency}}\>{\rm{CFA,}}\>{\rm{Day}}\>{\rm{7}}}}} \right)\; \times \;{\rm{100}} \cr} $$

Abbreviations: Average latency exp, Day 7 represents the average latency of the experimental group on Day 7, Average latency CFA, Day 7 represents the average latency of the CFA group on Day 7.

In addition, the Von Frey test was used to evaluate the sensitivity of the animals to mechanical pain. This test was performed before the administration of CFA and repeated on Days 1, 3, and 7 following the initiation of drug + CFA treatment for a total of four testing occasions. In brief, the mice were placed in a Von Frey testing acrylic enclosure (Ugo Basile, Varese, Italy) and allowed to become accustomed to the environment for 30 min. Then, Von Frey filaments were used in a specific central area at the bottom of the paws of the mice to measure the time to paw retraction. This procedure was repeated five times for each mouse, and the results were averaged for the analysis. Additionally, the final improvement rate for mechanical pain was calculated using the following formula (Eq. 3). The results are presented in Table 4.

**Table 4:** Mechanical pain improvement rate after 7 days of oral drug administration in CFA-injected mice

	Improvement rate of mechanical pain (%)
SBR	12.32%
Pel_0.5	11.09%
Pel_1.5	25.88%
SBR-Pel	37.50%

CFA: Complete Freund’s Adjuvant; Pel: Pelubiprofen; SBR: Shinbaro

3 $$\eqalign{& {\rm{Improvement rate of mechanical pain }}\left( {\rm{\% }} \right){\rm{ = }} \cr & \left( {{\matrix{{\rm{Average}}\>{\rm{latency}}\>{\rm{exp,}}\>{\rm{Day}}\>{\rm{7 - }} \hfill \cr {\rm{Average}}\>{\rm{latency}}\>{\rm{CFA,}}\>{\rm{Day}}\>{\rm{7}} \hfill \cr} \over {{\rm{Average}}\>{\rm{latency}}\>{\rm{CFA,}}\>{\rm{Day}}\>{\rm{7}}}}} \right)\;{\rm{ \times }}\;{\rm{10}} \cr} $$

Abbreviations: Average latency exp, Day 7 represents the average withdrawal latency of the experimental group on Day 7, Average latency CFA, Day 7 represents the average withdrawal latency of the CFA group on Day 7.

### Writhing test

Each drug was administered to the mice 1 h before acetic acid injection. Acetic acid was diluted to 0.7% with PBS. This solution (500 µL) was injected intraperitoneally into mice, and the writhing response was assessed for 30 min. Body elongation accompanied by contraction of the abdominal muscles was considered a writhing response.

### Real-time PCR

The changes in mRNA expression levels of *iNOS*, *CD86*, *IL-6*, *IL-1β*, *TNF-α*, *CD206*, *TGF-β*, and *IL-10* for each group were determined using quantitative PCR. mRNA was extracted using an RNeasy Fibrous Tissue Mini Kit (Qiagen, Hilden, Germany) and reverse transcribed using the oligo(dT)20 primer included in the AccuPower^®^ CycleScript™ RT PreMix (Bioneer, Korea). Real-time PCR was performed using an iQ SYBR Green Supermix (Bio-Rad, Hercules, CA, USA) on a CFX Connect Real-Time PCR Detection System (Bio-Rad). Gene expression was normalized to *GAPDH* levels. The results are reported as fold changes relative to the CFA group. The sequences of the primers used are listed in Table 5.

**Table 5:** Primer sequences used for real-time PCR analysis

Gene	5’-3’	Primer sequence
*iNOS*	Forward	CAGATCGAGCCCTGGAAGAC
	Reverse	CTGGTCCATGCAGACAACCT
*CD86*	Forward	TCAATGGGACTGCATATCTGCC
	Reverse	GCCAAAATACTACCAGCTCACT
*IL-6*	Forward	CCCCAATTTCCAATGCTCTCC
	Reverse	CGCACTAGGTTTGCCGAGTA
*IL-1β*	Forward	GAAATGCCACCTTTTGACAGTGA
	Reverse	GAAGGTCCACGGGAAAGACA
*TNF-α*	Forward	CAACCAACTAGTGGTGCCAG
	Reverse	ACTCCTCCCAGGTATATGGG
*CD206*	Forward	TTGGACGGATAGATGGAGGG
	Reverse	CCAGGCAGTTGAGGAGGTTC
*TGF-β*	Forward	TTGCTTCAGCTCCACAGAGA
	Reverse	TGGTTGTAGAGGGCAAGGAC
*IL-10*	Forward	TAACTGCACCCACTTCCCAG
	Reverse	AGGCTTGGCAACCCAAGTAA
*GAPDH*	Forward	GGCTCATGACCACAGTCCAT
	Reverse	TACTTGGCAGGTTTCTCCAGG

IL: interleukin; PCR: polymerase chain reaction; Pel: Pelubiprofen; SBR: Shinbaro; TNF: tumor necrosis factor; TGF-β: transforming growth factor beta

### Biochemical blood analysis

Biochemical analyses of blood profiles were conducted to assess the potential side effects of the drugs on liver and kidney function. Blood samples were collected from the retroorbital plexus while the animals were under anesthesia. Then, the samples were centrifuged at 3,000 rpm and 24 °C for 10 min to isolate the serum, which was analyzed using a DRI-CHEM NX600V analyzer (Fujifilm, Tokyo, Japan). This analysis used FDC slides for SGOT/ aspartate aminotransferase (AST), SGPT/alanine transaminase (ALT), alkaline phosphatase (ALP), creatinine (CR), and blood urea nitrogen (BUN) assays, in accordance with the manufacturer’s instructions. The AST, ALT, ALP, CR, and BUN levels were measured and reported as percentages.

### Statistical analyses

All numerical data are expressed as means ± standard errors of the mean. Comparisons among groups were performed using one-way analysis of variance (ANOVA) with Tukey’s post-hoc analysis (GraphPad Prism 8, Inc., La Jolla, CA, USA). Differences were considered statistically significant at ^#^*p* < 0.05, ^##^*p* < 0.01, ^###^*p* < 0.001, and ^####^*p* < 0.0001 vs. the blank or Sham group, ^*^p < 0.05, ^**^p < 0.01, ^***^p < 0.001, and ^***^p < 0.0001 vs. the LPS or CFA group, or ^&^*p* < 0.05, ^&&^*p* < 0.01, ^&&&^*p* < 0.001, and ^&&&&^*p* < 0.0001 vs. the SBR or Pel_0.5 group.

## Results

### Synergistic effects of SBR-Pel on enhancing cell viability and reducing cytotoxicity

**Fig. 1:**
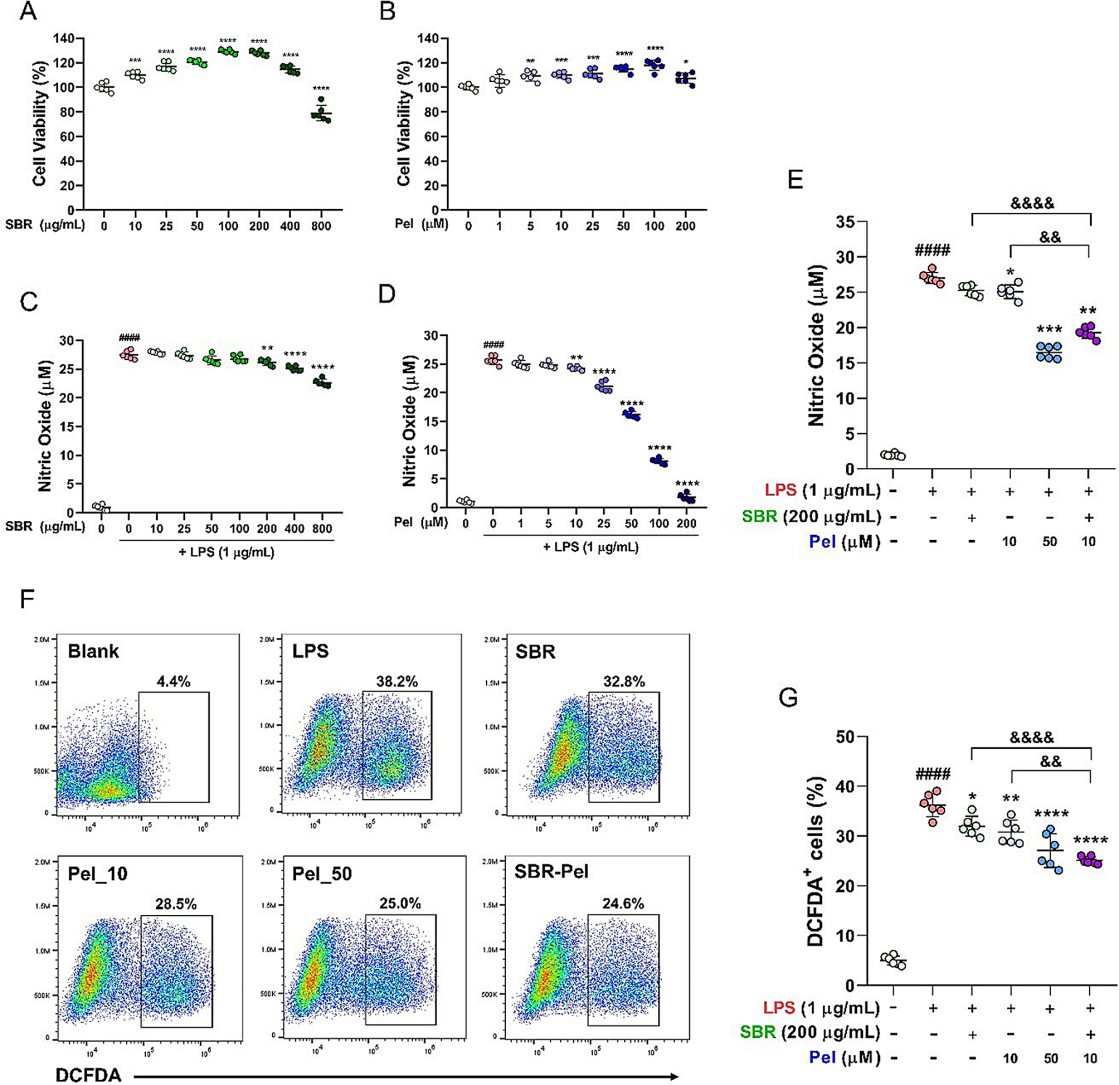
Effects of SBR and Pel on cell viability, NO, and ROS production in RAW264.7 macrophages. (**A**, **B**) Cell viability of RAW264.7 cells following treatment with varying concentrations of SBR (0–800 µg/mL) and Pel (0–200 µM) without LPS stimulation, assessed using the CCK assay. (**C**, **D**) NO production in LPS-stimulated cells after treatment with varying concentrations of SBR (0–800 µg/mL) and Pel (0–200 µM), assessed using the NO assay. (**E**) Comparative analysis of NO production in LPS-stimulated cells treated with the optimal concentrations of SBR (200 µg/mL) and Pel (10 or 50 µM) alone and in combination of SBR (200 µg/mL) and Pel (10 µM). (**F**, **G**) ROS levels in LPS-stimulated cells, assessed using DCFDA staining and quantified by flow cytometry. Data are expressed as means ± standard errors of the mean. Significant differences are indicated as ^####^*p* < 0.0001 vs. the blank group, ^*^p < 0.05, ^**^p < 0.01, ^***^p < 0.001, and ^****^p < 0.0001 vs. the LPS group, or ^&&^*p* < 0.01 and ^&&&&^*p* < 0.0001 vs. the SBR or Pel (10 µM) groups according to one-way analysis of variance with Tukey’s post-hoc test. CCK: cell counting kit; LPS: lipopolysaccharide; NO: nitric oxide; Pel: Pelubiprofen; ROS: reactive oxygen species; SBR: Shinbaro

To determine the optimal concentrations of SBR and Pel, we first established a range of concentrations that would not affect cell viability using the CCK assay 24 h after treating the cells with varying concentrations of SBR or Pel but without LPS. Our results showed that SBR was non-toxic to RAW264.7 cells at concentrations up to 200 µg/mL and even significantly improved cell viability. The pattern observed with SBR treatment alone showed a steady increase in cell viability up to 200 µg/mL. However, viability began to decrease at a concentration of 400 µg/mL, and significant toxicity was observed at 800 µg/mL (Fig. 1A). Conversely, Pel significantly enhanced cell viability without toxic effects up to a concentration of 100 µM. Starting at 200 µM, we observed a downward trend in cell viability (Fig. 1B). Then, we examined the effective dose of SBR or Pel required to inhibit NO production under LPS-treated conditions. When LPS was introduced to the cells, NO production significantly increased and exhibited a dose-dependent decreasing trend following SBR treatment, with a significant reduction observed at concentrations starting from 200 µg/mL (Fig. 1C). Under the same conditions, treatment with Pel elicited a concentration-dependent decrease in NO production from 1 to 200 µM. Notably, the suppression of NO production exhibited a significant and dramatic downward trend from 10 to 200 µM (Fig. 1D). Through these experiments, the combination treatment was assessed for its synergistic effects compared to individual treatments by setting the concentration at the lowest dose that showed significant inhibition of NO production: 200 µg/mL for SBR and 10 µM for Pel. Additionally, we evaluated the efficacy of the combination treatment against the effect observed at a higher concentration of Pel (10 µM) alone, specifically at 50 µM. NO production significantly increased following LPS treatment, whereas treatment with SBR or Pel alone led to a significant decrease in NO production compared with that in the LPS-treated group. Furthermore, the combination of SBR and Pel significantly reduced NO production (compared to the LPS group) and exhibited a significantly greater inhibitory effect on NO production than either SBR or Pel (10 µM) alone (Fig. 1E).

The ROS levels were measured using a dichlorodihydrofluorescein diacetate probe, and the results were imaged and quantified using flow cytometry (Fig. 1F, G). LPS treatment significantly increased ROS generation in RAW 264.7 cells. Compared with LPS treatment, treatment with SBR, two concentrations of Pel (10 and 50 µM), or a combination of SBR and Pel significantly reduced ROS levels. Notably, the combined treatment with SBR and Pel was the most effective in reducing ROS generation, showing a greater effect than either SBR or Pel (10 µM) alone. These findings show that a combination of SBR and Pel at concentrations optimized through the CCK and NO assays exhibits a synergistic effect, significantly enhancing the inhibition of NO and ROS production in LPS-treated RAW 264.7 cells more effectively than either compound alone. This highlights the potential of SBR-Pel as an anti-inflammatory agent.

### Synergistic effects of SBR-Pel on suppressing the release of pro-inflammatory cytokines and enhancing the expression of the macrophage mannose receptor, CD206, in LPS-stimulated RAW264.7 macrophages

**Fig. 2:**
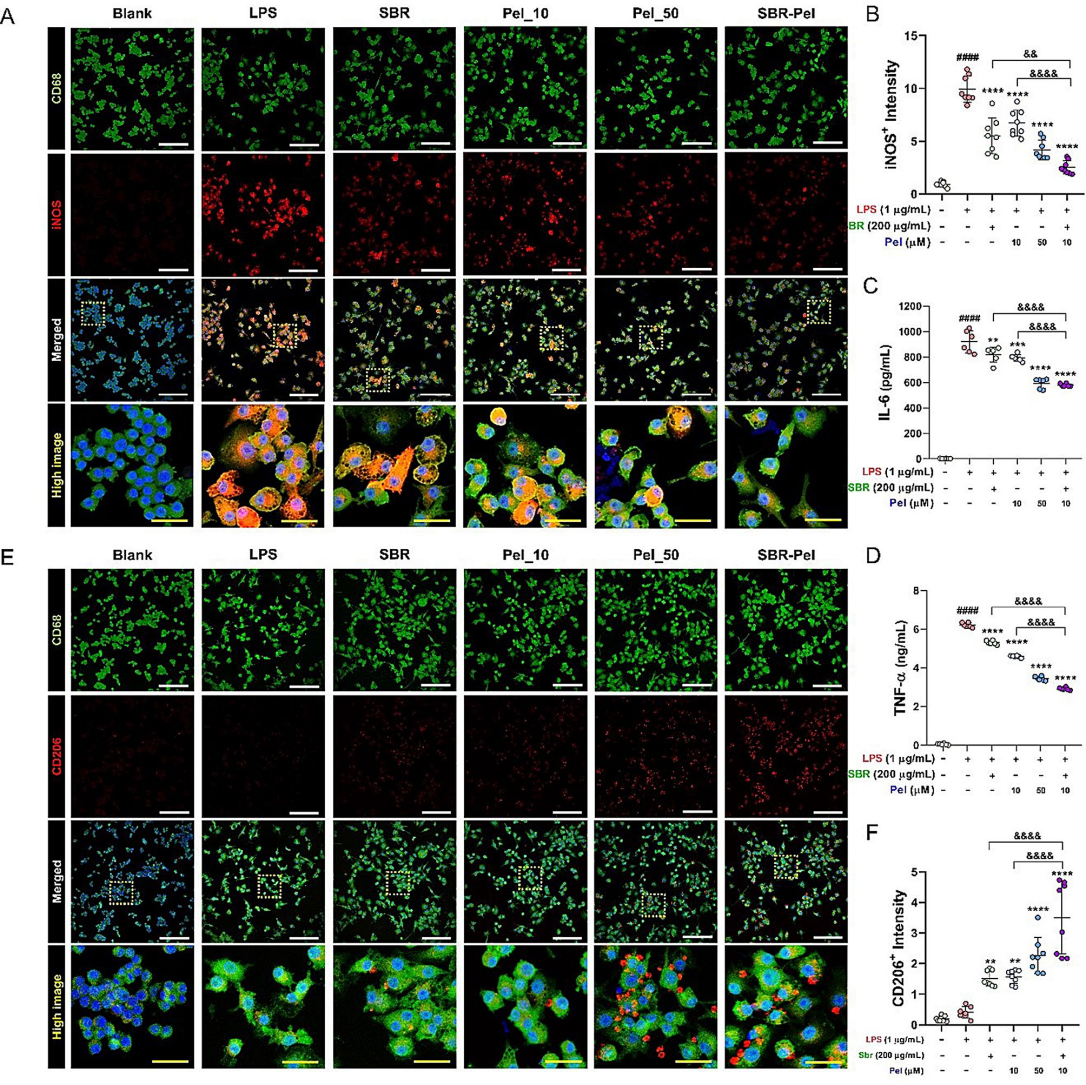
Impact of SBR-Pel on pro-inflammatory cytokine and mannose receptor CD206 expression in LPS-stimulated RAW264.7 macrophages. (**A**, **B**) Confocal images and graphs depict the visual data and quantitative evidence supporting iNOS activation. White or yellow scale bar = 150–30 μm. (**C**, **D**) ELISA of IL-6 and TNF-α activity from supernant in LPS-stimulated cells following treatment with SBR (200 µg/mL) and Pel (10 or 50 µM) alone or in combination. (**E**, **F**) Confocal images and graphs depict the visual data and quantitative evidence supporting CD206 activation. White or yellow scale bar = 150–30 μm. Data are expressed as means ± standard errors of the mean. Significant differences are indicated as ^####^*p* < 0.0001 vs. the blank group, ^**^p < 0.01, ^***^p < 0.001, and ^****^p < 0.0001 vs. the LPS group, or ^&^*p* < 0.05, ^&&^*p* < 0.01 and ^&&&&^*p* < 0.0001 vs. the SBR or Pel (10 µM) groups according to one-way analysis of variance with Tukey’s post-hoc test. IL: interleukin; Pel: Pelubiprofen; SBR: Shinbaro; TNF: tumor necrosis factor

The synergistic effect of the SBR-Pel combination on the expression of pro-inflammatory cytokines at the protein level was confirmed using immunocytochemistry and ELISA. Specifically, we examined the synergistic inhibitory effect on the expression of iNOS, IL-6, and TNF-α, which are typically secreted by M1 macrophages, following treatment with SBR-Pel. After LPS treatment, a marked increase in the expression of iNOS was observed, corroborating the role of LPS as a potent activator of M1 macrophage-mediated inflammation (Fig. 2A, B). Treatment with SBR or Pel (10 or 50 µM) alone resulted in a significant reduction in the iNOS^+^ intensity, indicating the anti-inflammatory potential of both agents. Notably, IL-6 and TNF-α activities, which are essential for the synthesis of pro-inflammatory mediators, also decreased following individual treatment with SBR or Pel (10 or 50 µM) (Fig. 2C, D). However, the combination of SBR and Pel exhibited a profound synergistic effect that significantly surpassed the anti-inflammatory action of either compound alone. This combination significantly reduced the levels of pro-inflammatory cytokines below those observed in the SBR or Pel (10 µM) groups. Moreover, this synergistic effect was evident in the substantial suppression of iNOS, IL-6, and TNF-α expression, highlighting the potential of SBR-Pel to target the enzymatic pathways involved in inflammation. Interestingly, the SBR-Pel combination also influenced the expression of CD206, a mannose receptor associated with the anti-inflammatory and healing activities of M2 macrophages. Compared with the SBR or Pel alone groups, the combination treatment significantly increased CD206 expression, suggesting an enhanced shift toward an anti-inflammatory macrophage phenotype (Fig. 2E, F). These findings suggest that the SBR-Pel combination not only effectively suppresses the secretion of pro-inflammatory mediators and enzymes involved in the inflammatory response, but also promotes an environment conducive to inflammation resolution and healing by enhancing CD206 expression. Therefore, the synergistic action of SBR and Pel holds significant promise for managing inflammation and its associated symptoms, offering a potential therapeutic strategy that targets both the reduction of pro-inflammatory signals and the promotion of anti-inflammatory mechanisms.

### Synergistic effects of SBR-Pel on attenuating CFA-induced paw edema in mice

**Fig. 3:**
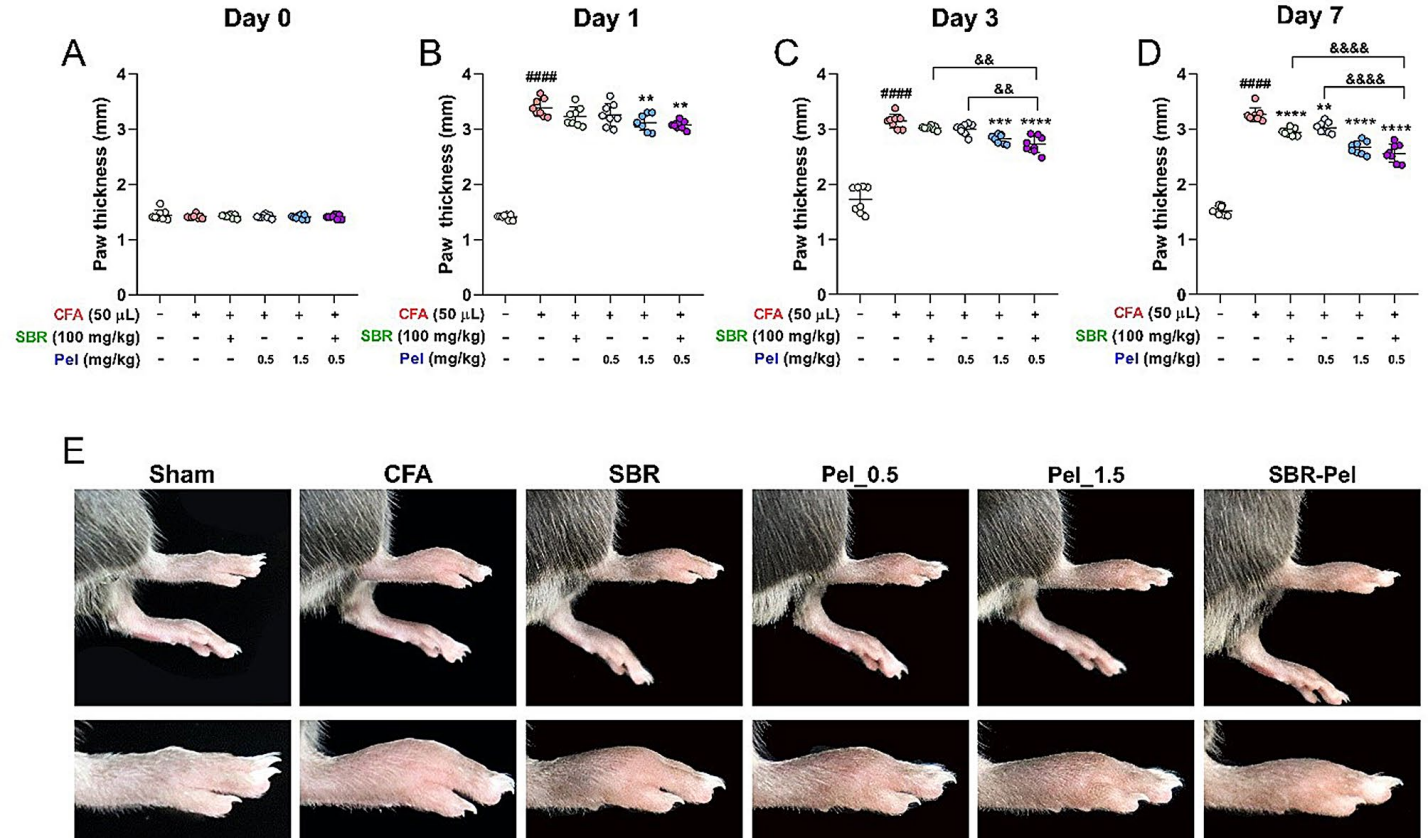
Evaluation of SBR-Pel’s anti-inflammatory effects on CFA-induced paw edema in mice. (**A**) Baseline paw thickness measurements across all groups prior to CFA administration on Day 0. (**B**) Paw thickness measurements on Day 1 following CFA and drug administration. (**C**) Paw thickness measurements on Day 3 following CFA and drug administration. (**D**) Paw thickness measurements on Day 7 following CFA and drug administration. (**E**) Images of paw tissues from all groups on Day 7. Data are expressed as means ± standard errors of the mean. Significant differences are indicated as ^####^*p* < 0.0001 vs. the Sham group, ^**^p < 0.01, ^***^p < 0.001, and ^****^p < 0.0001 vs. the CFA group, or ^&&^*p* < 0.01 and ^&&&&^*p* < 0.0001 vs. the SBR or Pel_0.5 groups according to one-way analysis of variance with Tukey’s post-hoc test. When calculating the final improvement rate for paw edema, the experimental group treated with the combination of SBR and Pel demonstrated an improvement rate of 21.39%. In contrast, the groups treated solely with SBR or Pel_0.5 exhibited improvement rates of 9.61% and 7.24%, respectively. These single-agent treatments resulted in markedly low improvement rates, below 10%, indicating minimal anti-inflammatory effects when used independently. Conversely, the SBR-Pel group exhibited a superior improvement rate exceeding 20% (Table 1). These results demonstrate the significant synergistic effect of SBR-Pel in alleviating paw edema induced by CFA. The reduction in paw thickness, especially on Days 3 and 7, along with histological evidence, underscores the potential of SBR-Pel as a therapeutic intervention for inflammation-related conditions. CFA: Complete Freund’s Adjuvant; Pel: Pelubiprofen; SBR: Shinbaro

The synergistic effect of SBR-Pel in alleviating paw edema was investigated by measuring paw thickness before and after the administration of CFA, as well as on Days 1, 3, and 7 after drug administration. We also measured the baseline paw thickness before administering CFA to ensure consistency across groups. The average paw thickness of the normal mice was 1.41 mm, with no significant differences between the groups, indicating a uniform baseline for the onset of CFA-induced inflammation (Fig. 3A). Following CFA administration, a noticeable increase in paw thickness was observed across all groups, indicating inflammation and edema formation, validating the inflammation model. A slight and statistically significant reduction in paw thickness was observed in the Pel_1.5 and SBR-Pel groups compared with that in the CFA group, suggesting the initiation of an anti-inflammatory effect (Fig. 3B). On Day 3 of drug administration, a significantly greater decrease in paw thickness was observed in the Pel_1.5 and SBR-Pel groups than in the CFA group, demonstrating a clear anti-inflammatory effect. Particularly, a significant reduction in paw thickness was observed in SBR-Pel group compared with that in the SBR or Pel_0.5 alone treatment group (Fig. 3C). When comparing the groups 7 days after the last dose, the SBR-Pel treatment group showed a superior reduction in paw edema compared with the CFA, SBR, or Pel_0.5 groups, highlighting the potential of SBR-Pel as an effective anti-inflammatory agent (Fig. 3D). Supporting the quantitative measurements taken on Day 7, images of paw from the SBR-Pel group showed an excellent reduction in edema, further confirming the anti-inflammatory efficacy of SBR-Pel (Fig. 3E). Analysis using H&E-stained images revealed that the paw thickness of the PBS-treated sham group showed no significant differences compared to normal mice across days 0, 1, 3, and 7. Similarly, the paw withdrawal latency measured by the von Frey test showed no significant differences between the two groups on any of the evaluated days (Figure S1A-C). To give a clearer view of the changes over time, we included images of the paw on days 0, 1, and 3, which visually illustrate the progression and reduction of edema, especially in the SBR-Pel group (Figure S1D).

### Synergistic effects of SBR-Pel on alleviating CFA-induced inflammation in mice

**Fig. 4:**
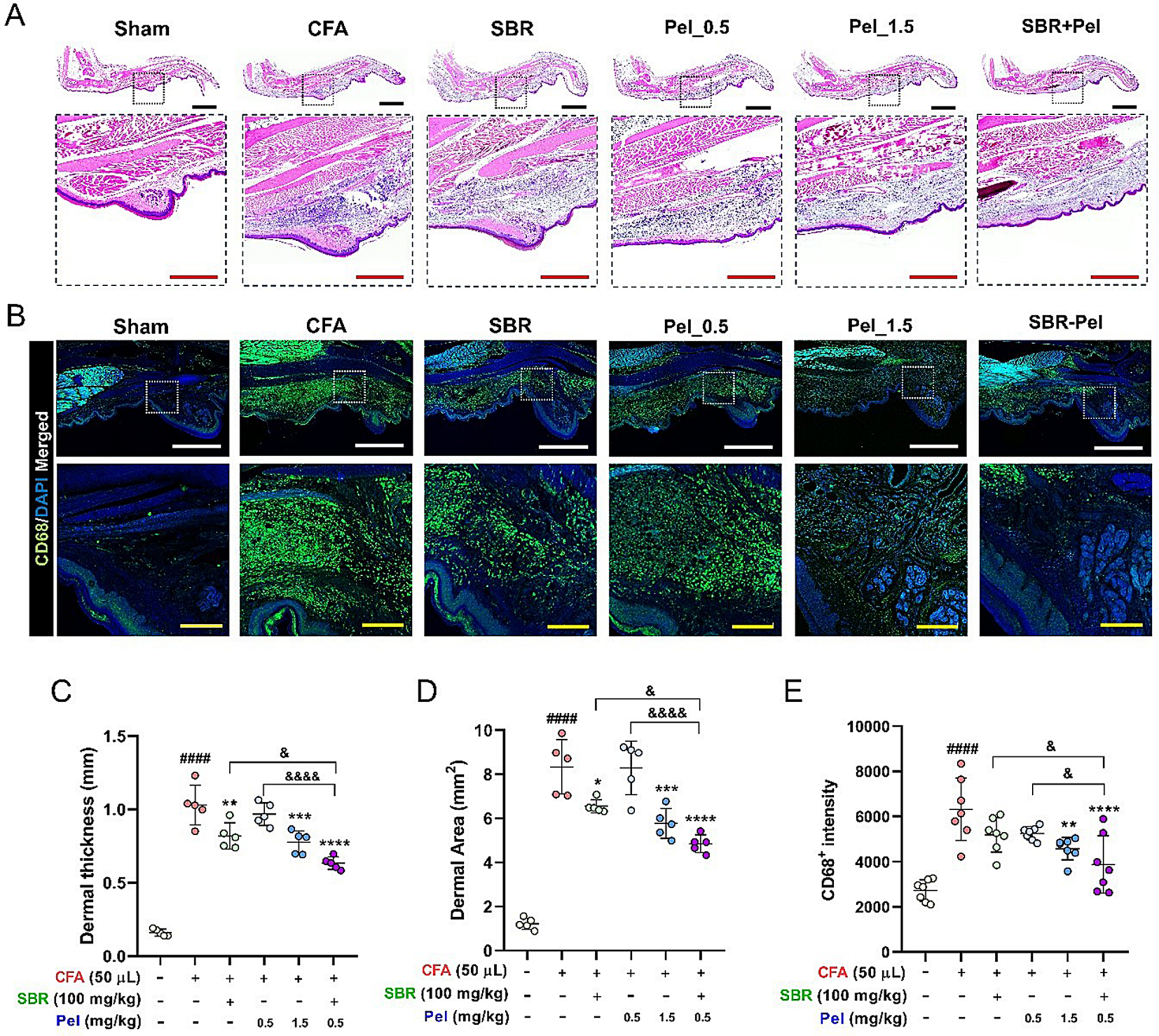
Histological analysis and immunostaining of rat paw sections following oral drug administration in CFA-injected mice. (**A**) Representative histological images using H&E staining display tissue responses at the plantar site of CFA injection. Black scale bar = 1 mm, Red scale bar = 500 μm. (**B**) CD68 immunostaining images illustrate macrophage infiltration at the site of inflammation induced by CFA injection. White scale bar = 500 μm, Yellow scale bar = 50 μm. (**C**, **D**) Graphical representations of the quantitative measurements of dermal thickness (mm) and area (mm^2^), respectively. (**E**) Quantitative analysis of CD68^+^ intensity across different treatment groups. Data are expressed as means ± standard errors of the mean. Significant differences are indicated as ^####^*p* < 0.0001 vs. the Sham group, ^**^p < 0.01, ^***^p < 0.001, and ^****^p < 0.0001 vs. the CFA group, or ^&^*p* < 0.05 and ^&&&&^*p* < 0.0001 vs. the SBR or Pel_0.5 groups according to one-way analysis of variance with Tukey’s post-hoc test

To further examine the synergistic effect of SBR-Pel in alleviating the inflammatory response induced by CFA injection on Day 7, we utilized histological techniques, applying both H&E staining and CD68 immunostaining to identify the macrophages and assess their status. H&E staining revealed increased swelling at the plantar site injected with CFA and clusters of hematoxylin-stained nuclei in the same area. Meanwhile, the images for both the SBR and Pel_0.5 groups demonstrated reduced swelling and inflammation caused by CFA injection. Notably, this reduction in the inflammatory response was more pronounced following treatment with Pel_1.5 or combined SBR-Pel, suggesting an enhanced effect (Fig. 4A). For the quantitative assessment, dermal thickness and area were measured and compared. Following CFA administration, there was a significant increase in these parameters. Notably, the SBR alone group exhibited a significant reduction compared with the CFA group. This reduction was even more marked in the Pel_1.5 and SBR-Pel combination groups. Among these, the SBR-Pel group showed the lowest dermal thickness and area, indicating a significant decrease when compared with that in both the SBR alone and Pel_0.5 alone groups (Fig. 4C, D). CD68 immunostaining revealed a significant increase in macrophage infiltration at the inflammation site following CFA injection compared with that in the Sham group. Notably, a marked reduction in macrophage infiltration was observed in the Pel_1.5 and SBR-Pel groups (Fig. 4B). Quantitative analysis of CD68 expression showed a significant elevation at the lesion sites following CFA administration. While the administration of SBR or Pel_0.5 alone tended to reduce CD68 expression levels, this decrease was not statistically significant. In contrast, the Pel_1.5 and SBR-Pel groups exhibited a significant reduction in CD68 expression, highlighting the efficacy of these treatments (Fig. 4E). These results indicated that combining SBR with a low concentration of Pel (0.5 mg/kg) yielded an anti-inflammatory effect comparable to that of high-concentration Pel (1.5 mg/kg) alone.

The improvement rate for CD68^+^ expression intensity in the group treated with the SBR-Pel combination was 38.59% (Table 2). In contrast, the improvement rates for the groups treated solely with SBR or Pel_0.5 were 17.94% and 16.89%, respectively. The single-agent groups showed very low improvement rates, below 20%, indicating minimal effectiveness when administered independently. Conversely, the group receiving the combined SBR-Pel treatment exhibited a significantly superior improvement rate, exceeding 35%.

CFA: Complete Freund’s Adjuvant; Pel: Pelubiprofen; SBR: Shinbaro.

### Synergistic effects of SBR and Pel on modulating mRNA levels of pro-inflammatory and anti-inflammatory cytokines in the footpad skin of CFA-injected mice

**Fig. 5:**
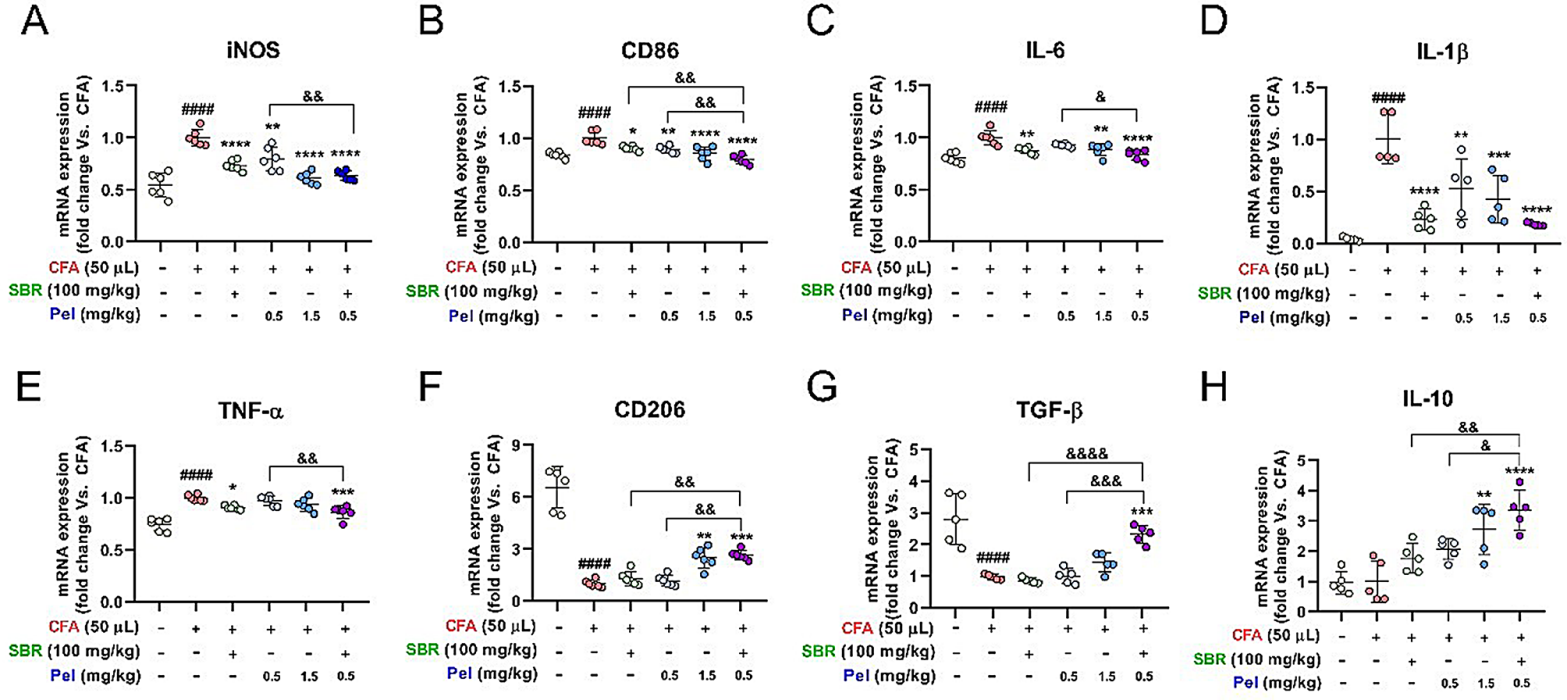
mRNA expression of pro-inflammatory and anti-inflammatory cytokines in CFA-injected mice treated with therapeutic agents. (**A**–**E**) Graphs showing the mRNA levels of pro-inflammatory cytokines (*iNOS*, *CD86*, *IL-6*, *IL-1β*, and *TNF-α*) in the footpad skin of mice injected with CFA and administered with therapeutic agents. (**F**–**H**) Graphs depicting the mRNA levels of anti-inflammatory cytokines (*CD206*, *TNF-β*, and *IL-10*) in response to treatment. Data are expressed as means ± standard errors of the mean. Significant differences are indicated as ^####^*p* < 0.0001 vs. the Sham group, ^*^p < 0.05, ^**^p < 0.01, ^***^p < 0.001, and ^****^p < 0.0001 vs. the CFA group, or ^&^*p* < 0.05, ^&&^*p* < 0.01, ^&&&^*p* < 0.001 and ^&&&&^*p* < 0.0001 vs. the SBR or Pel_0.5 groups according to one-way analysis of variance with Tukey’s post-hoc test. CFA: Complete Freund’s Adjuvant; IL: interleukin; Pel: Pelubiprofen; SBR: Shinbaro; TNF: tumor necrosis factor

Next, we examined whether the synergistic anti-inflammatory effects observed in histological analyses were associated with changes in the mRNA levels of pro-inflammatory and anti-inflammatory cytokines. We observed a significant increase in the mRNA expression of pro-inflammatory cytokines after administering CFA, including *iNOS*, *CD86*, *IL-6*, *IL-1β*, and *TNF-α*, as measured using real-time PCR (Fig. 5A–E). Conversely, the groups treated with the therapeutic agents exhibited a significant reduction in the mRNA levels of these cytokines. Particularly, the combination of SBR and Pel markedly decreased the levels of these cytokines more effectively than the other treatments. The reduction in *iNOS*, *CD86*, *IL-6*, and *TNF-α* mRNA levels was significantly greater in the SBR and Pel combination group than in the group treated with a low dose of Pel (0.5 mg/kg). This effect was as strong as, or stronger than, that observed in the group treated with a higher dose of Pel (1.5 mg/kg). Furthermore, drug treatment influenced the regulation of cytokines related to anti-inflammatory responses. The combination of SBR and Pel upregulated the expression of anti-inflammatory cytokines, including *CD206*, *TNF-β*, and *IL-10*, more significantly than individual treatment (Fig. 5F–H). This increase suggests that the SBR-Pel treatment promoted stronger anti-inflammatory responses than any single agent alone, indicating a robust anti-inflammatory effect.

CFA: Complete Freund’s Adjuvant; IL: interleukin; Pel: Pelubiprofen; SBR: Shinbaro; TNF: tumor necrosis factor.

### Evaluation of liver and kidney toxicity of SBR-Pel combination

**Fig. 6:**
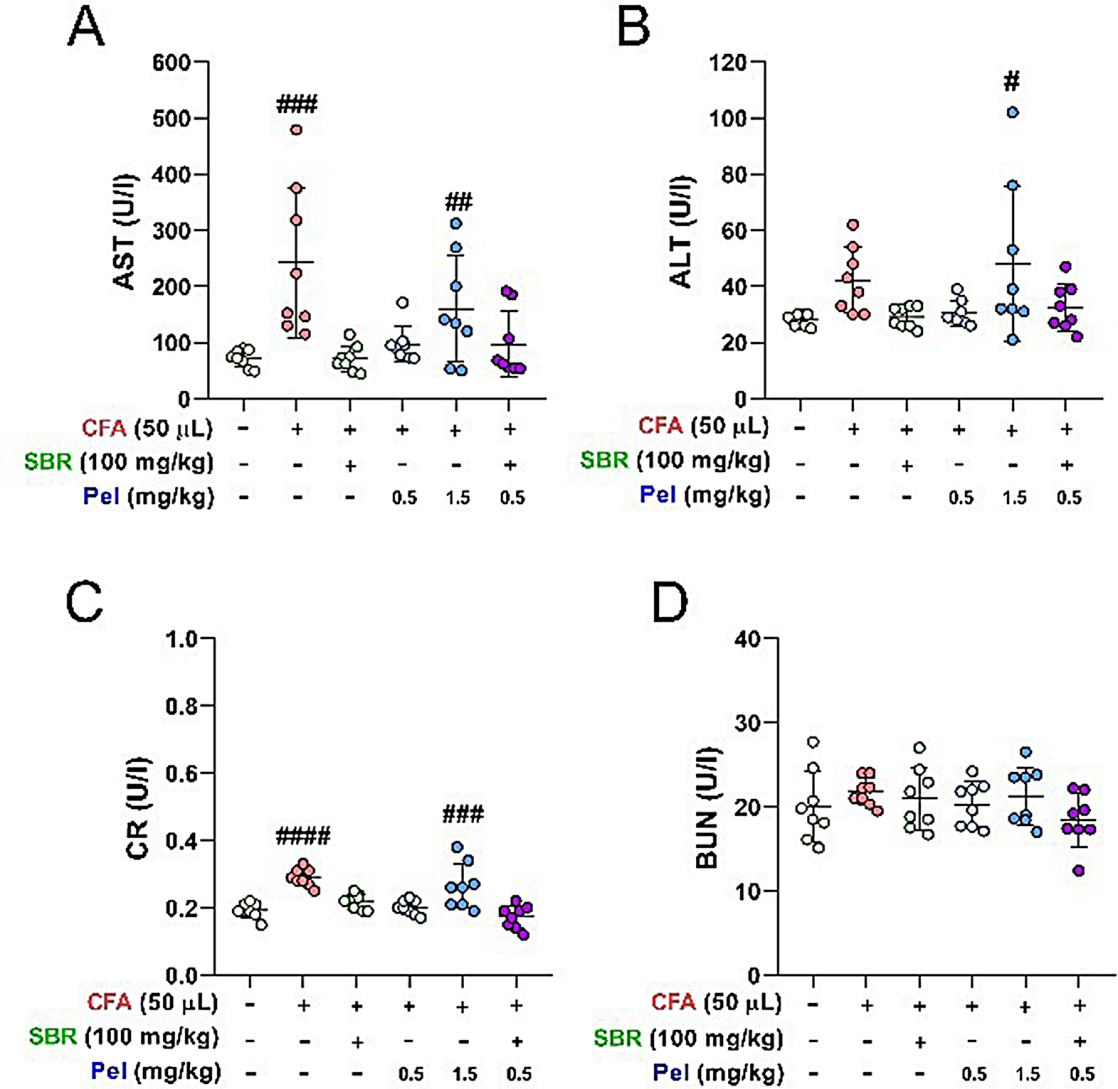
Blood Biochemical Assays for Evaluating Liver and Kidney Toxicity of the SBR-Pel Combination in CFA-Injected Mice. (**A**, **B**) Graphs displaying liver enzyme levels (AST and ALT) in blood serum. (**C**, **D**) Graphs showing the creatinine (CR) and blood urea nitrogen (BUN) levels, indicators of kidney function. Data are expressed as means ± standard errors of the mean. Significant differences are indicated as ^####^*p* < 0.0001 vs. the Sham group, ^**^p < 0.01, ^***^p < 0.001, and ^****^p < 0.0001 vs. the CFA group, or ^&&^*p* < 0.01 and ^&&&&^*p* < 0.0001 vs. the SBR or Pel groups according to one-way analysis of variance with Tukey’s post-hoc test

Pel is known to increase liver enzyme levels and BUN as side effects. To investigate whether the combination of SBR and Pel could alleviate these side effects in comparison to administering high doses of Pel alone (Choi et al. 2014), we evaluated liver toxicity-related AST and ALT levels in blood serum, and checked the CR and BUN levels for kidney toxicity after CFA injection. At 7 days post CFA injection, the AST levels were significantly higher than those of the Sham group. The group treated with 1.5 mg/kg of Pel also showed a significant increase in both AST and ALT levels compared with the Sham group. However, the combination group that included SBR and 0.5 mg/kg Pel exhibited liver enzyme levels similar to those of the Sham group, with no significant increase observed (Fig. 6A, B). Additionally, regarding hepatotoxicity, there were no significant differences between groups in serum ALP levels or in the mRNA expression of liver pro-inflammatory cytokines (*IL-6*, *TNF-α*, and *IL-1β*) as assessed by real-time PCR (Figure S2A-D). Furthermore, histopathological examination of liver, kidney, and lung tissues using H&E staining showed no signs of tissue toxicity in mice, with an injury score of 0 for all evaluated organs. Additionally, there were no significant differences in organ indices (liver, kidney, and lung) between the groups (Figure S2E-J). Furthermore, the CR levels, an indicator of kidney toxicity, significantly increased post CFA injection in the Pel_1.5 group compared with that in the Sham group. In contrast, the remaining groups, including the SBR-Pel combination group, showed CR levels similar to those of the Sham group. There was no significant difference in the BUN levels among the groups. These findings suggest that combining SBR with low concentrations of Pel may mitigate the risk of liver or kidney toxicity associated with high concentrations of Pel (Fig. 6C, D).

### Synergistic analgesic effects of SBR-Pel combination on CFA-induced inflammatory pain

**Fig. 7:**
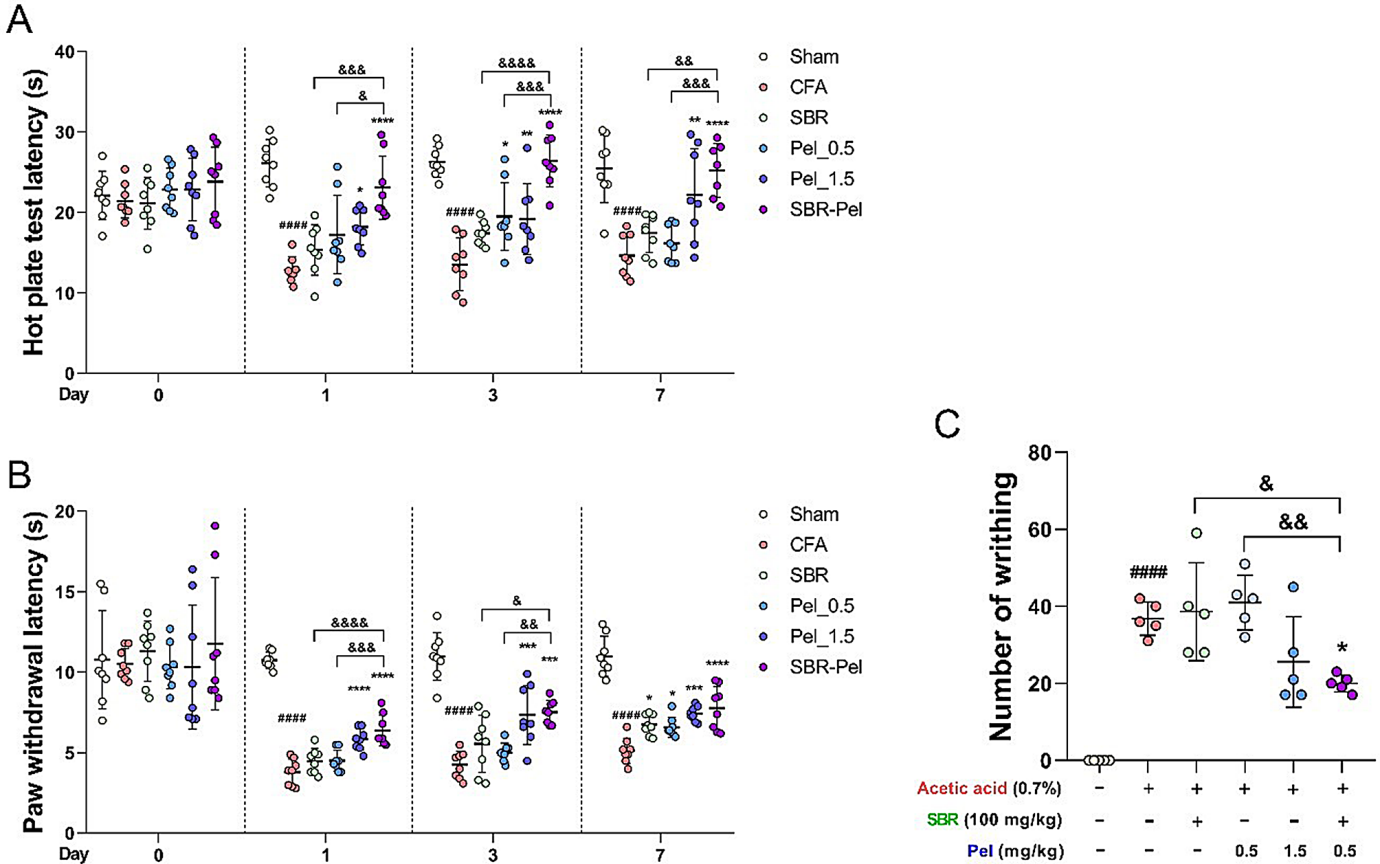
Nociceptive assays of inflammatory pain responses in CFA-injected mice treated with therapeutic agents. (**A**) Results from the hotplate test demonstrating the effects of treatments on thermal hyperalgesia before and on Days 1, 3, and 7 post CFA injection. (**B**) Results from the Von Frey test illustrating changes in withdrawal latency to mechanical stimuli before and on Days 1, 3, and 7 post-CFA injection. (**C**) Graph displaying the number of writhing reactions 1 h after drug administration, induced by intraperitoneal injection of acetic acid. Data are expressed as means ± standard errors of the mean. Significant differences are indicated as ^####^*p* < 0.0001 vs. the Sham group, ^**^p < 0.01, ^***^p < 0.001, and ^****^p < 0.0001 vs. the CFA group, or ^&&^*p* < 0.01 and ^&&&&^*p* < 0.0001 vs. the SBR or Pel groups according to one-way analysis of variance with Tukey’s post-hoc test

Finally, to confirm the synergistic improvement effect of SBR-Pel on inflammatory pain, we conducted a hotplate test for thermal hyperalgesia. Measurements were taken before CFA injection and then, on Days 1, 3, and 7 after the initiation of drug administration, averaging the results over these periods. Compared with SBR alone group, the SBR-Pel group showed significant improvements in thermal hyperalgesia from day 1, indicating a significant synergistic effect, which continued to increase through Day 7, showing significant differences compared with the SBR or Pel 0.5 alone groups on Days 3 and 7 (Fig. 7A). Additionally, the synergistic suppressive effect of SBR-Pel on pain sensitivity to mechanical stimuli was assessed using the Von Frey test. From Day 1, the Pel_1.5 and SBR-Pel groups showed a significant increase in withdrawal latency compared with the CFA group, while on Day 3, the SBR-Pel group exhibited a significantly higher latency than the Pel_0.5 alone group. However, by Day 7, all groups showed an increasing trend in latency with no significant differences between them (Fig. 7B). Finally, we assessed the analgesic effects of the drugs on acute pain by measuring the number of writhing responses following intraperitoneal injection of acetic acid, which induces painful reactions and acute inflammatory contractions in the abdomen. Upon administration of the SBR-Pel combination, we observed a significant reduction in the number of writhing compared with that in the CFA group and the groups treated with SBR alone or Pel_0.5 alone (Fig. 7C). Overall, combining SBR with low Pel concentrations offers a promising approach for managing inflammatory pain while minimizing liver and kidney toxicity associated with high Pel doses.

The improvement rate for thermal pain in the group treated with the combination of SBR and Pel was 49.53% (Table 3), while the rates for the groups treated individually with SBR or Pel_0.5 were 20.16% and 13.65%, respectively. Therefore, compared with the single treatment groups, the combination of SBR and Pel exhibited an improvement effect nearly reaching 50%.

AST: aspartate aminotransferase; ALT: alanine aminotransferase; BUN: blood urea nitrogen; CR: creatinine; CFA: Complete Freund’s Adjuvant; Pel: Pelubiprofen; SBR: Shinbaro.

Similarly, as detailed in Table 4, the improvement rate for mechanical pain in the group treated with the combination of SBR and Pel was 37.50%. In contrast, the improvement rates for the groups treated individually with SBR or Pel_0.5 were 12.32% and 11.09%, respectively. While the single treatment groups showed very low improvement rates of < 15%, the SBR-Pel combination group demonstrated a significant improvement rate exceeding 35% for mechanical hyperalgesia.

## Discussion

This study examined the synergistic anti-inflammatory and analgesic effects of SBR and Pel, highlighting a potential integrated approach for inflammation management. Our findings demonstrated a significant reduction in paw swelling and edema, leading to effective pain relief through a synergistic decrease in inflammatory cytokine expression and CD68^+^ macrophage infiltration within inflamed tissues after SBR-Pel injection. These results suggest that SBR-Pel is a promising candidate for new combination treatments that improve efficacy and minimize side effects, thus, paving the way for further exploration of the therapeutic potential of SBR-Pel and its role in anti-inflammatory therapy.

Previous studies have demonstrated that both agents independently showed efficacy in reducing inflammation. SBR, which comprises herbal ingredients, such as Gucheok, Bangpung, Useul, Ohgapi, Duchung, and Heukdu, inhibits pro-inflammatory mediators like COX-2, TNF-α, MMP-2, and MMP-9. Pel, an NSAID, blocks the COX pathway, reducing prostaglandin synthesis and the production of inflammatory factors, such as NO and TNF-α (Lee et al. 2011). Despite its benefits, SBR may cause allergies or digestive issues in some individuals (Jung et al. 2019), whereas the long-term use of Pel can lead to gastrointestinal, cardiovascular, and renal side effects (Choi et al. 2014). To minimize the side effects of treatment and maximize effectiveness, combining natural substances with pharmaceutical drugs offers multiple advantages. This strategy might yield a synergistic effect, enhance treatment outcomes, and potentially reduce the required dosage of each component, thereby lowering the risk of adverse effects.

Natural substances typically include a range of bioactive compounds, providing a broad therapeutic action that can bolster the targeted effects of conventional drugs (Ding and Xue 2024; Nasim et al. 2022; Ahn et al. 2023). Our findings underscore the advantages of combining SBR and Pel. Initial CCK analyses identified the non-cytotoxic concentration ranges for both compounds, with SBR enhancing cell viability at concentrations up to 200 µg/mL and Pel up to 100 µM. Notably, cell viability exceeding 100% suggests that treatment with optimal concentrations of SBR or Pel promoted cell viability, growth, or metabolic activity beyond baseline levels.

Further investigation revealed a dose-dependent decrease in NO production by both SBR and Pel, with their combination (SBR [200 µg/mL] and Pel [10 µM]) showing a greater reduction than either compound alone, indicating synergistic effects. In addition, the combined treatment significantly lowered ROS production in LPS-induced RAW264.7 macrophages, surpassing the effects of either compound alone. This indicates a synergistic enhancement of the antioxidant capacity of either agent, which was further supported by the significant decrease in NO production elicited by the combined treatment. These findings suggest that the synergistic combination of SBR and Pel improves cell viability and significantly reduces the cellular toxic effects induced by pro-inflammatory stimuli. The SBR-Pel combination offers strategic advantages by potentially lowering the required therapeutic dose of each compound, thereby minimizing side effects associated with higher doses.

To determine the optimal concentration combination of SBR and Pel for in vivo experiments, we referred to existing patents and research literature. SBR was shown to exhibit anti-inflammatory effects starting at a concentration of 100 mg/kg, while Pel was reported to suppress inflammation and pain beginning at 0.5 mg/kg. Considering the potential for a synergistic effect, we opted for a combination of SBR at 100 mg/kg and Pel at 0.5 mg/kg to maximize efficacy at lower concentrations (Yoon et al. 2023). Additionally, for the high-concentration Pel group, we used 1.5 mg/kg, which is the next concentration level reported in the literature following 0.5 mg/kg. In vivo results demonstrated the superior anti-inflammatory potential of the SBR-Pel combination, which significantly reduced paw swelling in CFA-injected mice and modulated the pro- and anti-inflammatory cytokine levels. M1 macrophages are pivotal in the initial phase of the inflammatory response, secreting pro-inflammatory cytokines (iNOS, CD86, IL-6, IL-1β, and TNF-α) that mediate the defense against pathogens and initiate tissue repair. Conversely, M2 macrophages play a crucial role in resolving inflammation and promoting tissue healing through the secretion of anti-inflammatory cytokines (CD206, TNF-β, and IL-10). Our results indicate that the SBR-Pel combination effectively modulates this balance by reducing M1-associated pro-inflammatory cytokine production while enhancing M2-associated anti-inflammatory cytokine secretion, suggesting a mechanism for its anti-inflammatory efficacy. Additionally, the results suggested that SBR-Pel could amplify the analgesic responses to CFA-induced inflammatory pain without causing liver or kidney toxicity, indicating the therapeutic potential of this combination in managing inflammation and related discomfort.

However, this study has several limitations. Firstly, it focused on acute inflammation models, which may not fully represent chronic inflammatory processes. Furthermore, the long-term effects, safety, and synergistic effects of the SBR-Pel combination on chronic inflammation have not been investigated. Secondly, we did not elucidate the specific pathways and interactions related to the combined efficacy of SBR and Pel. Therefore, future research should include an in-depth molecular mechanism analysis of the complementary synergistic effects of the SBR-Pel combination, as well as investigate the therapeutic potential and safety for long-term use in chronic inflammatory diseases.

## Conclusions

Through both in vitro and in vivo experiments, we demonstrated the synergistic anti-inflammatory effects of SBR-Pel, which include the reduction of NO, ROS, and pro-inflammatory cytokine levels. Additionally, SBR-Pel effectively reduced paw edema and lowered macrophage infiltration and pro-inflammatory cytokine expression in inflamed tissues induced by CFA. Interestingly, SBR-Pel also enhanced the expression of anti-inflammatory cytokines, indicating a balanced immune response. Furthermore, our results highlight the potential of SBR-Pel in alleviating inflammatory pain while reducing liver and kidney toxicity associated with high Pel doses. This suggests that SBR-Pel could be a promising treatment option for inflammatory conditions driven by macrophage activity, offering improved effectiveness and fewer side effects.

## Electronic supplementary material

Below is the link to the electronic supplementary material.

Supplementary Material 1

Scheme 1

Scheme 2

## Data Availability

No datasets were generated or analysed during the current study.

## Declarations

### Abbreviations

AST: aspartate aminotransferase;

ALT: alanine aminotransferase;

BUN: blood urea nitrogen;

CR: creatinine;

CFA: Complete Freund’s Adjuvant;

CCK-8: Cell Counting Kit-8;

ELISA: Enzyme-linked immunosorbent assay;

H&E: hematoxylin and eosin;

IL: interleukin;

LPS: lipopolysaccharide;

NO: nitric oxide;

PBS: phosphate-buffered saline;

Pel: Pelubiprofen;

SBR: Shinbaro;

TNF: tumor necrosis factor;
