# Supplementary material for: Synergistic attenuation of complete freund’s adjuvant-induced inflammation in mice using shinbaro-pelubiprofen: a novel therapeutic complex
Source: Mol Med. 2025 Jan 21;31:17. doi: 10.1186/s10020-025-01083-y (PMC11753103; doi:10.1186/s10020-025-01083-y)
Supplement: Supplementary file 1 — Supplementary Material 1 [file 10020_2025_1083_MOESM1_ESM.docx]

**
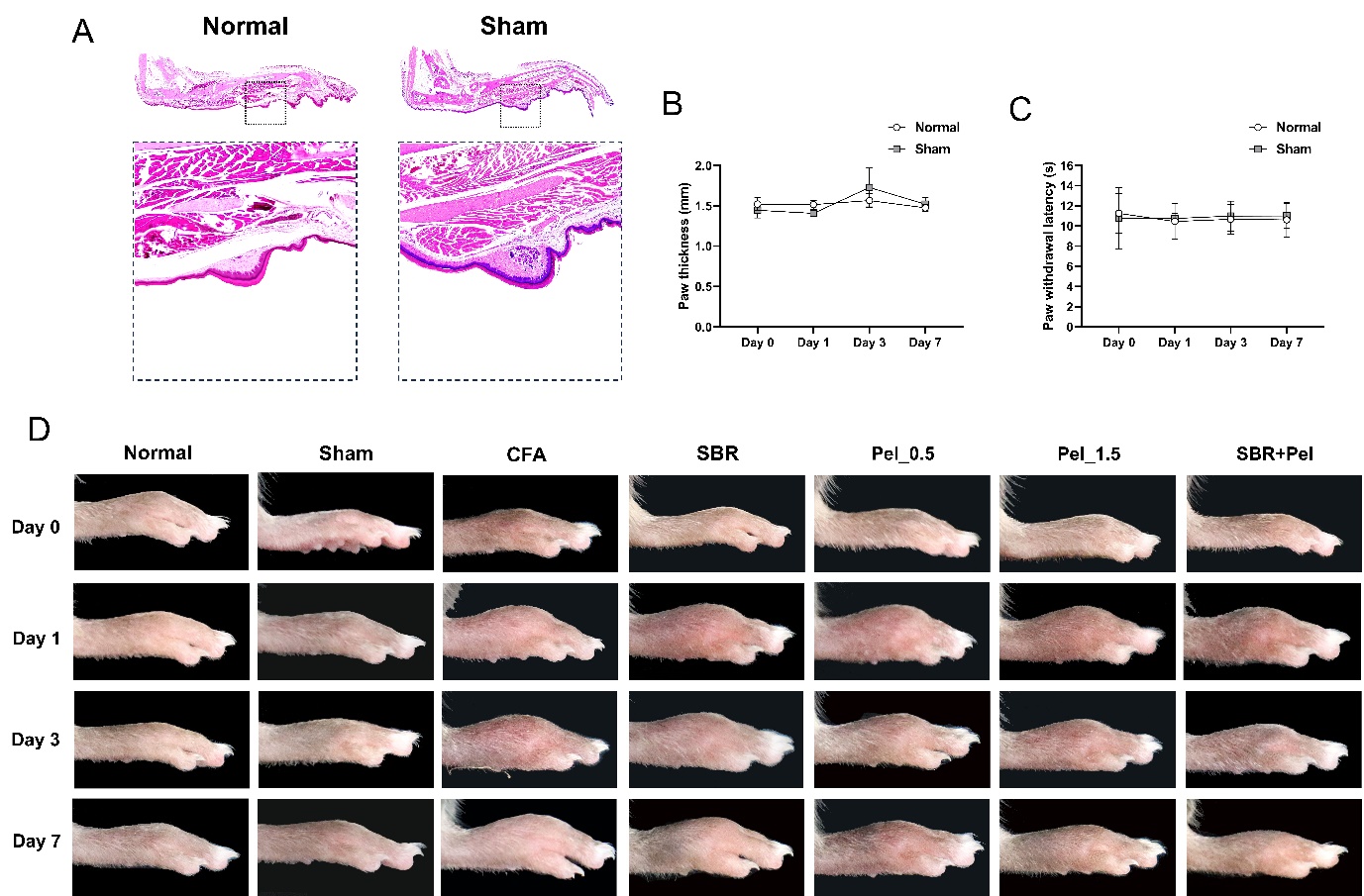
Figure S1. (**A) Representative H&E images of the normal and sham groups, illustrating the morphology of the paw tissue. (B) Quantitative graph of paw thickness (mm) measured on days 0, 1, 3, and 7 in the normal and sham groups. (C) Quantitative assessment of paw withdrawal latency (s) measured by von Frey test on days 0, 1, 3, and 7 in the normal and sham groups. (D) Time-course images of paw edema in each group. Representative images of the paw on days 0, 1, 3, and 7 are shown to illustrate the progression and reduction of edema over time. Data are expressed as means ± standard errors of the mean.

**
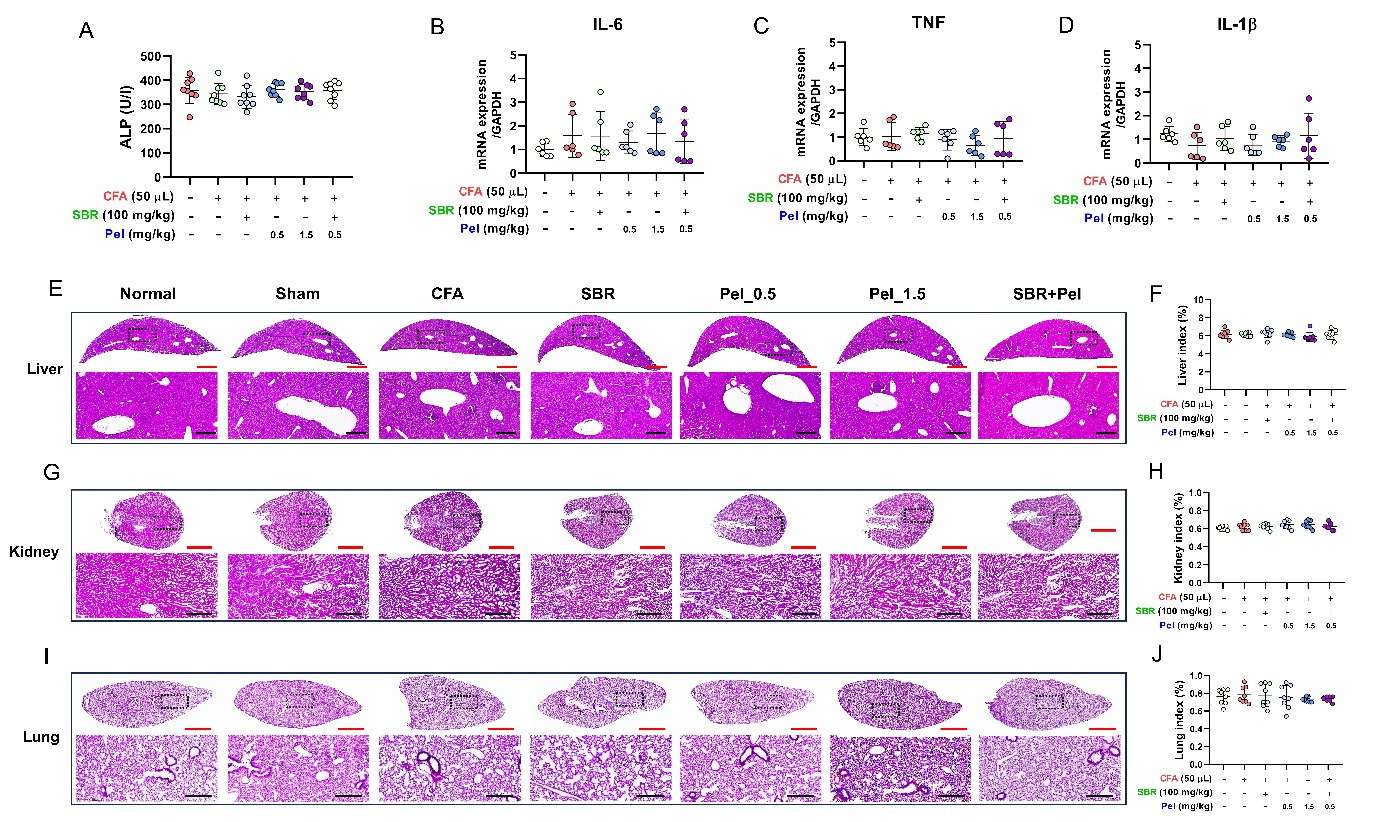
Figure S2. Hepatotoxicity and histopathological assessment of liver, kidney, and lung tissues.** (A) Graphs displaying serum ALP levels in each group. (B-D) Relative mRNA expression levels of liver pro-inflammatory cytokines: *IL-6* (B), *TNF-α* (C), and *IL-1β* (D), analyzed by real-time PCR. (E) Representative H&E-stained images of liver tissue from each group, illustrating histopathological features. (F) Liver index (%) calculated as (liver weight/body weight) × 100. (G) Representative H&E-stained images of kidney tissue from each group (H) Kidney index (%) calculated as (kidney weight/body weight) × 100. (I) Representative H&E-stained images of lung tissue from each group. (J) Lung index (%) calculated as (lung weight/body weight) × 100. Red Scale bar = 2 mm. Black Scale bar = 400 μm. Data are expressed as means ± standard errors of the mean.
