# Supplementary figures and images for: Synergistic attenuation of complete freund’s adjuvant-induced inflammation in mice using shinbaro-pelubiprofen: a novel therapeutic complex
Source: Mol Med. 2025 Jan 21;31:17. doi: 10.1186/s10020-025-01083-y (PMC11753103; doi:10.1186/s10020-025-01083-y)

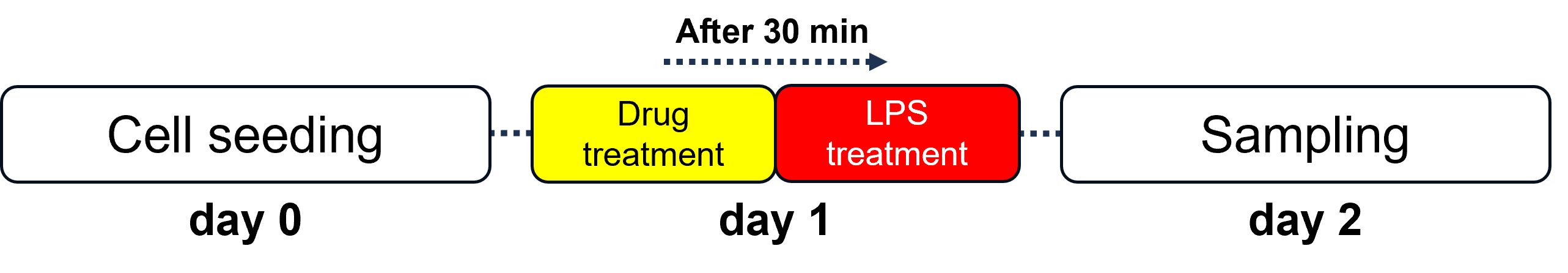

Supplement: Supplementary file 2 — Scheme 1 [file 10020_2025_1083_MOESM2_ESM.png]

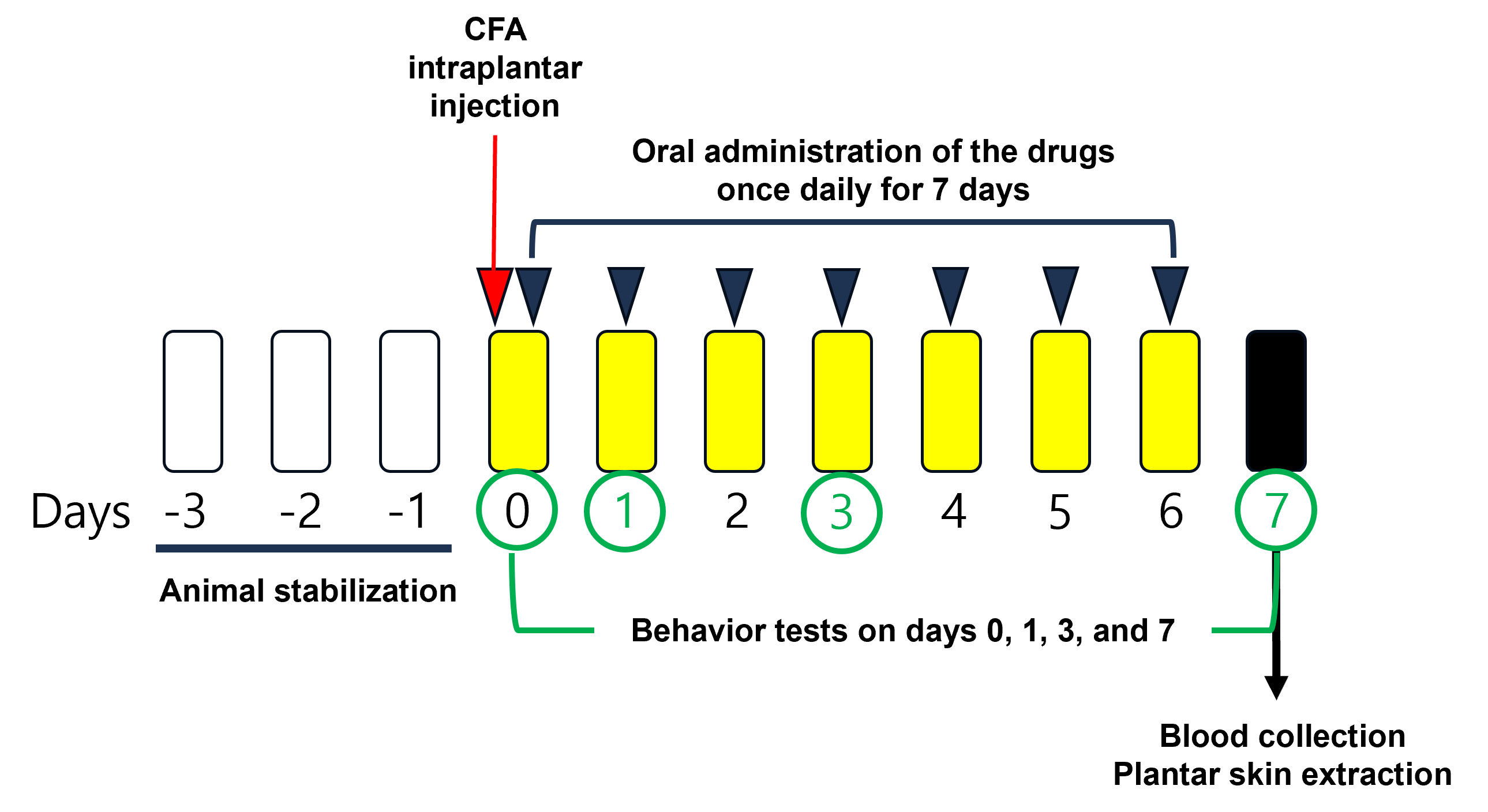

Supplement: Supplementary file 3 — Scheme 2 [file 10020_2025_1083_MOESM3_ESM.png]
